# Current MitraClip experience, safety and feasibility in the Netherlands

**DOI:** 10.1007/s12471-017-0992-1

**Published:** 2017-04-25

**Authors:** Z. Rahhab, F. A. Kortlandt, J. F. Velu, R. A. J. Schurer, V. Delgado, P. Tonino, A. J. Boven, B. J. L. Van den Branden, A. O. Kraaijeveld, M. Voskuil, J. Hoorntje, M. van Wely, K. van Houwelingen, G. B. Bleeker, B. Rensing, I. Kardys, J. Baan jr., J. A. S. Van der Heyden, N. M. Van Mieghem

**Affiliations:** 1 Department of Cardiology, Thorax center, Erasmus Medical Center, Rotterdam, The Netherlands; 2 Department of Cardiology, St. Antonius Hospital, Nieuwegein, The Netherlands; 3 Department of Cardiology, Academic Medical Center, Amsterdam, The Netherlands; 4 Department of Cardiology, University Medical Center Groningen, Groningen, The Netherlands; 5 Department of Cardiology, Leiden University Medical Center, Leiden, The Netherlands; 6 Department of Cardiology, Catharina Hospital, Eindhoven, The Netherlands; 7 Department of Cardiology, Zorggroep Noorderbreedte, Leeuwarden, The Netherlands; 8 Department of Cardiology, Amphia Hospital, Breda, The Netherlands; 9 Department of Cardiology, University Medical Center Utrecht, Utrecht, The Netherlands; 10 Department of Cardiology, University Medical Center Maastricht, Maastricht, The Netherlands; 11 Department of Cardiology, Radboud University Medical Center, Nijmegen, The Netherlands; 12 Department of Cardiology, Medisch Spectrum Twente, Twente, The Netherlands; 13 Department of Cardiology, Haga Hospital, The Hague, The Netherlands

**Keywords:** Valvular heart disease, Mitral valve, Mitral valve therapies

## Abstract

**Purpose:**

Data on MitraClip procedural safety and efficacy in the Netherlands are scarce. We aim to provide an overview of the Dutch MitraClip experience.

**Methods:**

We pooled anonymised demographic and procedural data of 1151 consecutive MitraClip patients, from 13 Dutch hospitals. Data was collected by product specialists in collaboration with local operators. Effect on mitral regurgitation was intra-procedurally assessed by transoesophageal echocardiography. Technical success and device success were defined according to modified definitions of the Mitral Valve Academic Research Consortium (MVARC).

**Results:**

Median age was 76 (interquartile range 69–82) years and 59% were males. Patients presented with ≥moderate mitral regurgitation and a predominance of functional mitral regurgitation (72%). Overall, 611 (53%) patients were treated with one Clip, 486 (42%) with ≥2 Clips and 54 (5%) received no Clip. The number of patients with ≥2 Clips increased from 22% in 2009 to 52% in 2016. Device success and technical success were 91 and 95%, respectively, and were consistent over the years. Significant reduction of mitral regurgitation by MitraClip was achieved in 94% of patients and was observed more often in patients with functional mitral regurgitation (95% vs. 91%, *p* = 0.025). Device time declined from 145 min in 2009 to 55 min in 2016.

**Conclusion:**

MitraClip experience in the Netherlands is growing with excellent technical success and device success. Over the years, device time decreased and more patients were treated with ≥2 Clips.

## Introduction

Mitral regurgitation (MR) has a 2% prevalence in the general population and is more frequent in the elderly [1, 2]. Surgical treatment is considered the ‘gold standard’ for patients with symptomatic severe mitral regurgitation [3]. However, a significant proportion (49%) of eligible patients are denied for surgery because of age, comorbidities or poor left ventricular function [4].

The MitraClip (Abbott Vascular, Menlo Park, CA) offers a completely percutaneous mitral valve edge-to-edge repair. The efficacy and safety of the MitraClip device have been demonstrated in the EVEREST I (Endovascular Valve Edge-to-Edge Repair Study) trial [5]. Subsequently, the EVEREST II randomised controlled trial compared conventional surgery with MitraClip in operable patients with moderate-to-severe or severe, predominantly degenerative MR [6]. MitraClip was associated with superior safety and similar improvements in clinical outcomes. However, it was less effective in reducing MR [6]. Based on these results, the Food and Drug Administration approved MitraClip for high-risk patients with symptomatic degenerative MR. In European practice, the majority of patients treated with MitraClip have functional MR [7, 8]. In this clinical setting, MitraClip may improve survival and hospital readmissions [9].

Data on the Dutch MitraClip experience are scarce. We therefore aim to provide an informative overview of the current MitraClip procedural safety and efficacy in the Netherlands.

## Methods

This multicentre observational retrospective study collected all patients (n = 1151) from 13 Dutch hospitals treated with MitraClip between January 2009 and June 2016. All patients were discussed in local multi-disciplinary heart teams including interventional cardiologists, imaging specialist and cardiac surgeons, and were considered symptomatic and at high operative risk. All patients provided written informed consent for the MitraClip procedure.

Procedural data were prospectively and anonymously collected by product specialists in collaboration with local operators and, after approval of the participating centres, retrospectively analysed. Effect on MR was intra-procedurally (onsite) assessed by transoesophageal echocardiography. The Medical Ethics Committee of the Erasmus Medical Center reviewed the study protocol and waived the need for additional informed consent because of the non-interventional character of this retrospective study (MEC-2016-423) using anonymous data collection. The investigation conforms to the principles outlined in the Declaration of Helsinki.

### MitraClip procedure

**Fig. 1:**
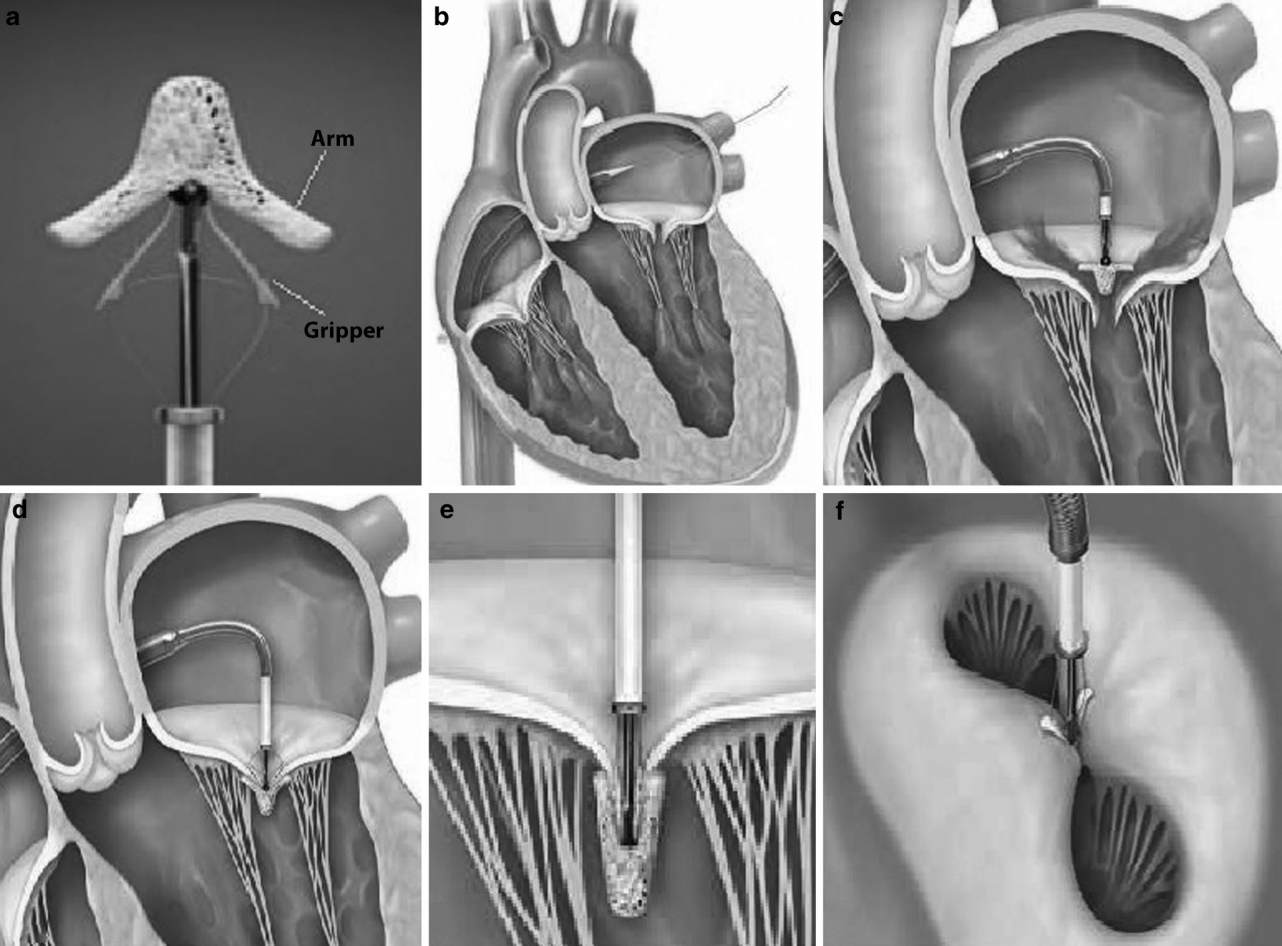
**a** MitraClip device with two movable arms and grippers; **b** Guide catheter advanced into the left atrium after transseptal puncture; **c** Positioning of the MitraClip above the regurgitation jet perpendicular to mitral coaptation line; **d** The MitraClip is pulled back in order to capture both leaflets; **e** The grippers are lowered and the arms are closed approximating the leaflets; and **f** creating a double mitral orifice. Image courtesy of Abbott

The MitraClip device is a 4 mm wide, polyester-covered cobalt chromium V‑shaped clip with two movable arms and grippers (Fig. 1a). All procedures are performed under general anaesthesia, using fluoroscopic and transoesophageal echocardiographic guidance. A 24-French guide catheter is introduced in a femoral vein and delivered into the left atrium after transseptal puncture (Fig. 1b). The clip delivery system is advanced through the guide catheter into the left atrium and positioned above the origin of the regurgitation jet, perpendicular to the mitral coaptation line (Fig. 1c). The arms of the Clip are opened and advanced into the left ventricle. The Clip is then gradually pulled back towards the left atrium in order to grasp both mitral valve leaflets (Fig. 1d). The grippers are lowered, the clip is closed (Fig. 1e) and the leaflets are approximated resulting in a double mitral orifice (Fig. 1f). Before releasing the Clip, the severity of MR is assessed and the transmitral gradient is measured. If the result is satisfactory, the Clip can be released. In case of inadequate MR reduction or a high transmitral gradient, the Clip can be opened and repositioned or removed. More than 1 Clip may be necessary for significant MR reduction.

### Study endpoints and definitions

The primary endpoints were procedural safety expressed in ‘technical success’ and procedural efficacy expressed in ‘device success’, both were modified from the Mitral Valve Research Consortium (MVARC) criteria [10].

- Technical success is defined as successful deployment of the device with absence of procedural mortality and freedom from emergency surgery.
- Device success is defined as proper placement of the device without procedural mortality and with reduction in post-procedural MR by ≥1 grade from baseline and to an absolute level of ≤moderate MR.
- Significant MR reduction: reduction in post-procedural MR by ≥1 grade from baseline.
- Device time is defined as the time from guide catheter insertion to guide catheter removal.

### Statistical analysis

Categorical variables are presented as frequencies and percentages, and compared with the use of the Pearson Chi Square Test or the Fisher’s exact test, as appropriate. Continuous variables are presented as means (± standard deviation − SD), in case of normal distribution, or medians (interquartile range − IQR), in case of skewed distribution, and compared with the use of the Student’s t‑test or the Mann-Whitney U test. Normality of the distributions was assessed using the Shapiro-Wilk test. We used a two-sided alpha level of 0.05 to indicate significance. Statistical analyses were performed using SPSS software version 21.0 (SPSS Inc., Chicago, Illinois, USA).

## Results

**Fig. 2:**
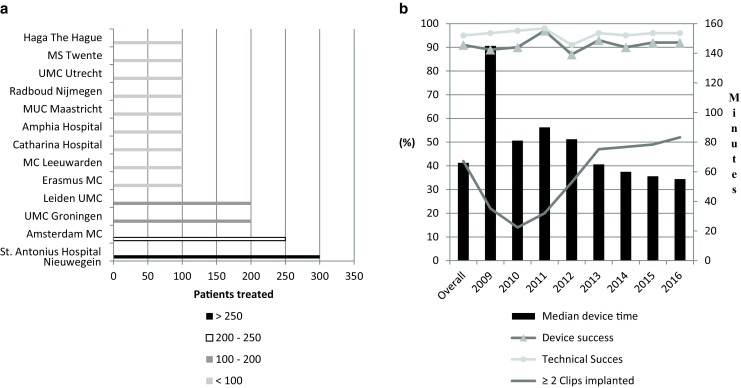
Overview of **a** the relative contributions of the participating centres and **b** procedural characteristics and the primary endpoints over the years

A total of 1151 patients underwent percutaneous mitral valve edge to-edge repair with the MitraClip device. Relative contributions of the participating centres are summarised in Fig. 2a. The overall cohort had a median age of 76 (IQR 69–82) years and 59% were males. All patients presented with ≥moderate MR at baseline, with a clear dominance of functional MR (72%) (Table 1). Overall, 611 (53%) patients were treated with one Clip, 486 (42%) with ≥2 Clips and 54 (5%) received no Clip (Table 2). The number of patients treated with ≥2 Clips increased from 22% in 2009 to 52% in 2016 (Fig. 2). Significant MR reduction (≥1 grade) was achieved in 94% of patients.

**Table 1:** Baseline characteristics of patients undergoing MitraClip implantation

	Total population 2009–2016
	(*n* = 1151)
Male, *n* (%)	684 (59)
Age, median (IQR)	76 (69–82)
*Etiology MR*	
Degenerative, *n* (%)	198 (17)
Functional, *n* (%)	832 (72)
Mixed, *n* (%)	118 (10)
Unknown, *n* (%)	3 (0.3)
*Severity of MR at baseline*	
Moderate, *n* (%)	19 (2)
Moderate-to-severe, *n* (%)	388 (34)
Severe, *n* (%)	744 (65)
LVEF <30%, *n* (%)	500 (43)

*IQR* interquartile range, *MR* mitral regurgitation*, LVEF* left ventricular ejection fraction

**Table 2:** Procedural characteristics of patients undergoing MitraClip implantation

	Total population 2009–2016
	(*n* = 1151)
*Clips*	
0 Clips, *n* (%)	54 (5)
1 Clip, *n* (%)	611 (53)
≥2 Clips, *n* (%)	486 (42)
Device Time (min)^a^, median (IQR)	66 (42–103)
*MR reduction*	
0, *n* (%)	75 (7)
1, *n* (%)	108 (9)
2, *n* (%)	587 (51)
3, *n* (%)	381 (33)
≥1, *n* (%)	1076 (94)
Device success^b^, *n* (%)	1049 (91)
Technical success^c^, *n* (%)	1097 (95)
Intra-procedural death, *n* (%)	3 (0.3)
Emergency surgery, *n* (%)	6 (0.5)

*IQR* interquartile range, *MR* mitral regurgitation

^a^Device time: defined as the time from delivery system insertion to clip delivery system removal

^b^Device success: defined as proper placement of the device without procedural mortality and with reduction in

post-procedural MR by ≥1 grade from baseline and to an absolute level of ≤moderate MR

^c^Technical success: defined as successful deployment of the device with absence of procedural mortality

and freedom from emergency surgery

The overall device and technical success were 91 and 95%, respectively, and were consistent over the years (Fig. 2b). Intra-procedural death and need for emergency surgery occurred in 3 (0.3%) and 6 (0.5%) patients, respectively (Table 2). The median device time declined from 145 (IQR 108–177) minutes in 2009 to 55 (IQR 34–86) minutes in 2016 (Fig. 2b).

### Degenerative vs. functional MR

**Fig. 3:**
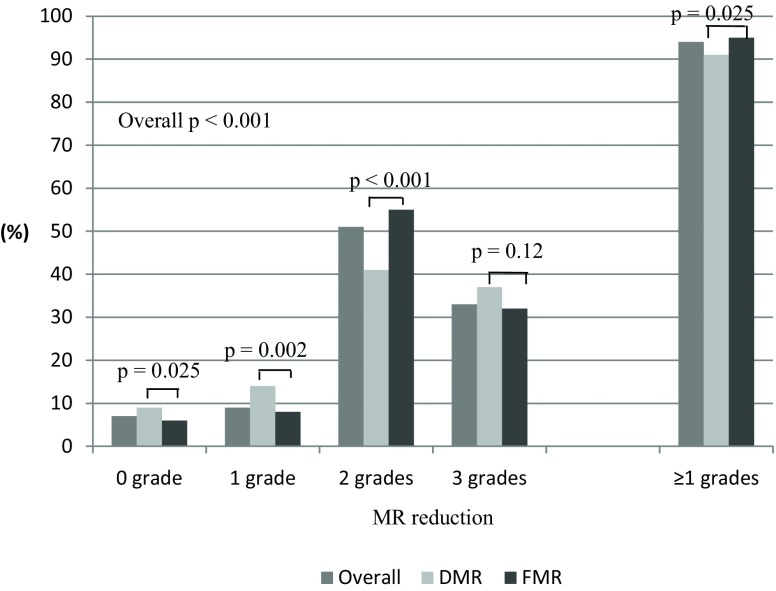
Comparison of reduction of mitral regurgitation in patients with degenerative mitral regurgitation versus functional mitral regurgitation. *MR* mitral regurgitation*, DMR* degenerative mitral regurgitation*, FMR* functional mitral regurgitation

Patients with degenerative MR were older (median age 82 [IQR 76–85] vs. 74 [IQR 67–79] years, *p* < 0.001), had more often severe MR at baseline (73% vs. 61%, *p* < 0.001) and were more often treated with ≥2 Clips (50% vs. 39%, *p* = 0.001) when compared to patients with functional MR. Patients in the latter group had more often significant MR reduction (95% vs. 91%, *p* = 0.025) (Fig. 3) and a shorter device time (62 [IQR 40–99] minutes vs. 75 [IQR 49–110] minutes, *p* < 0.001).

### One vs. ≥two MitraClips

**Fig. 4:**
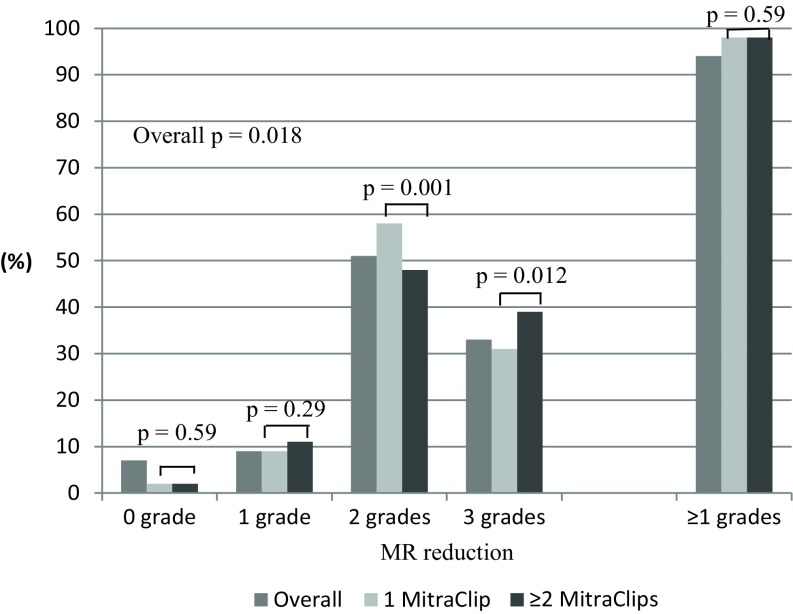
Comparison of mitral regurgitation reduction in patients treated with 1 versus ≥2 MitraClips. *MR* mitral regurgitation

Patients treated with ≥2 Clips were more often males (68% vs. 53%, p < 0.001) with degenerative MR (33% vs. 23%, *p* < 0.001) and severe MR at baseline (81% vs. 53%, *p* < 0.001). Significant MR reduction was similar in both groups (98% vs. 98%, *p* = 0.59) (Fig. 4) while median device time was higher in ≥2 Clips group (86 [IQR 58–120] vs. 51 [IQR 35–75] minutes, *p* < 0.001).

## Discussion

To date, more than 1250 patients have undergone MitraClip treatment in the Netherlands. We present the largest Dutch multi-centre MitraClip study including 1151 patients. Key findings are: 1) MitraClip was predominantly used to treat functional MR; 2) MitraClip was successful in reducing MR in 94% of patients; 3) MitraClip was slightly more effective in patients with functional MR; 4) Over the years, implantation of ≥2 Clips became more frequent; 5) With growing experience, procedure time decreased with preserved device success and technical success.

Patient demographics in our study were comparable with large European registries (i. e. ACCESS-Europe A Two-Phase Observational Study of the MitraClip System in Europe (ACCESS-EU) and German Transcatheter Mitral Valve Interventions Registry [TRAMI]) but different from the EVEREST-II trial. The EVEREST trial was conducted in the USA and included younger patients (67.3 ± 12.8 years) with preserved left ventricular ejection fraction (60 ± 10.1). In Europe, MitraClip is more often applied in functional MR, which contrasts with the clear dominance (73%) of degenerative MR in the USA (Table 3; [6–8]).

**Table 3:** Baseline and procedural characteristics of patients undergoing MitraClip implantation in different cohorts

	MitraClip Netherlands	ACCESS-EU Phase I	German TRAMI Registry	EVEREST-II
	(*n* = 1151)	(*n* = 567)	*n* = 1064	*n* = 184
Male, *n* (%)	684 (59)	362 (64)	658 (62)	115 (63)
Age (years)	76 (69–82)	73.7 ± 9.6	75 (70–81)	67.3 ± 12.8
*Etiology MR*				
Degenerative, *n* (%)	198 (17)	117 (23)	246 (29)	135 (73)
Functional, *n* (%)	832 (72)	393 (77)	590 (71)	49 (27)
Mixed, *n* (%)	118 (10)	**–**	**–**	**–**
Unknown, *n* (%)	3 (0.3)	**–**	**–**	**–**
*Severity of MR at baseline*				
Moderate, *n* (%)	19 (2)	13 (2)	42 (5)	8 (4)
Moderate-to-severe, *n* (%)	388 (34)	230 (41)	**–**	130 (71)
Severe, *n* (%)	744 (65)	324 (57)	827 (95)	46 (25)
LVEF <30%, *n* (%)	500 (43)	193 (34)	294 (33)	N. A.
LVEF, mean ± SD	N. A.	N. A.	N. A.	60 ± 10.1
*Procedural*				
0 Clips, *n* (%)	54 (5)	2 (0.4)	N. A.	N. A.
1 Clip, *n* (%)	611 (53)	(60)	N. A.	N. A.
≥2 Clips	486 (42)	(40)	N. A.	N. A.
*Severity of MR after Clip*				
≤moderate, *n* (%)	1057 (92)	475 (91)	417 (97)	(77)
Moderate-to-severe, *n* (%)	57 (5)	39 (8)	–	41 (23)
Severe, *n* (%)	37 (3)	7 (1)	17 (3)	–
Device success^a^, *n* (%)	1049 (91)	N. A.	N. A.	N. A.
Technical success^b^, *n* (%)	1097 (95)	N. A.	N. A.	N. A.
Intra-procedural death, *n* (%)	3 (0.3)	0 (0)	0 (0)	N. A.
Emergency surgery, *n* (%)	6 (0.5)	N. A.	N. A.	N. A.

*MR* mitral regurgitation, *LVEF* left ventricular ejection fraction, *SD* standard deviation

^a^Device success: defined as proper placement of the device without procedural mortality and with reduction in post-procedural MR by ≥1 grade from baseline and to an absolute level of ≤moderate MR

^b^Technical success: defined as successful deployment of the device with absence of procedural mortality and freedom from emergency surgery

In our study, MitraClip seemed slightly more effective in functional MR than in degenerative MR (95% vs. 91%, *p* = 0.025). Intra-procedural death and ≤moderate MR after Clip implantation were comparable with the ACCESS-EU and TRAMI registry (0.3% vs. 0% vs. 0% and 92% vs. 91% vs. 97%, respectively), confirming the safety and efficacy of MitraClip (Table 3).

Over the years, practice changed with a higher frequency of implanting ≥2 Clips. Patients treated with ≥2 Clips were more often males with degenerative MR and severe MR at baseline. Patients with degenerative MR may have thicker and more mobile leaflets and had (in our cohort) more often severe MR at baseline, which may explain why these patients in particular are treated with ≥2 Clips. A previous study identified anterior leaflet thickness (OR 1.7 per mm [95% CI; 1.16–2.57], *p* = 0.007) and a greater regurgitation volume at baseline (OR 1.21 per 10 ml [95% CI; 1.0–1.3], *p* = 0.01) as echocardiographic predictors for the need for more than 1 Clip [11]. Another study showed that the vena contracta (jet width) predicted need for >1 Clip (OR 2.5 [95% CI; 1.2–5.3], *p* = 0.013) with 83% sensitivity and 90% specificity for a cut-off value of ≥7.5 mm [12]. The increased device time in degenerative MR may also be explained by thicker and more mobile leaflets since this may aggravate the grasping process. Another reason is simply because of implantation of more Clips.

According to the latest European guidelines on valvular heart disease, MitraClip may be considered in patients with symptomatic severe primary and secondary MR, despite optimal medical therapy, including cardiac resynchronization therapy, who fulfil the echo criteria of eligibility, are judged inoperable or at high surgical risk by a heart team, and have a life expectancy greater than 1 year (recommendation Class IIb, level of evidence C) [3]. The American guidelines consider transcatheter mitral valve repair only for severely symptomatic patients with chronic severe primary MR who have favourable anatomy for the repair procedure and a reasonable life expectancy, but who have a prohibitive surgical risk because of severe comorbidities and remain severely symptomatic despite optimal guideline-directed medical therapy for heart failure (recommendation Class IIb, level of evidence B) [13]. Yet, a wealth of recent clinical data underscores procedural safety and efficacy of MitraClip and a favourable longer-term outcome in selected patients. MitraClip seems an excellent treatment strategy in patients who are deemed at very high or prohibitive operative risk by heart team consensus. Several studies have shown significant MR reduction in the vast majority of high-risk patients, resulting in positive left ventricular remodelling and improvement of functional capacity [14, 15].

Also, heart failure patients who do not respond effectively to cardiac resynchronisation therapy and have at least moderate MR can improve with MitraClip. Auricchio et al. showed that 73% of cardiac resynchronization therapy non-responders (with functional MR) improved in functional class, and had increased left ventricular ejection fraction and reduced ventricular volumes after MitraClip treatment [16].

Ongoing randomised trials further elaborate on the value of MitraClip in functional MR. The MATTERHORN (Mitral vAlve reconsTrucTion for advancEd Insufficiency of Functional or iscHaemic ORigiN) trial, is comparing MitraClip with reconstructive mitral valve surgery in high-risk patients with moderate-to-severe functional MR. The Cardiovascular Outcomes Assessment of the MitraClip Percutaneous Therapy for Heart Failure Patients with Functional Mitral Regurgitation (COAPT) trial is investigating the safety and efficacy of MitraClip versus optimal medical treatment (OMT) in patients with moderate-to-severe or severe functional MR who have been assessed as not eligible for mitral valve surgery.

The Multicentre Study of Percutaneous Mitral Valve Repair MitraClip Device in Patients With Severe Secondary Mitral Regurgitation (MITRA-FR) is comparing the safety, efficacy and the cost-effectiveness of OMT versus OMT plus MitraClip in patients with severe secondary mitral regurgitation.

Expectedly, focused guidelines on valvular heart disease will be updated in the foreseeable future and include stronger recommendations for MitraClip. For now, our study demonstrated substantial MitraClip experience in the Netherlands with excellent procedural safety and efficacy.

## Limitations

Given the retrospective observational character of this study and the onsite assessment of MR (i. e. absence of echo core lab), potential self-reporting bias may be introduced. Specific echocardiographic (quantitative) parameters such as regurgitation volume and jet width were not available. In addition, data were limited to procedural outcome. Follow-up data are needed to evaluate the durability of device success.

Long-term efficacy may reveal recurrence of MR (grade 3 or 4) as shown by the EVEREST-II trial and ACCESS-EU study with more than moderate MR recurrence rates of 21% at 12 months in both studies. Furthermore, we also acknowledge that complications such as stroke, bleeding and vascular complications, although rare, may occur during follow-up.

## Conclusion

MitraClip experience in the Netherlands is growing with excellent technical success and device success. Over the years, the device time decreased and more patients were treated with ≥2 Clips.
